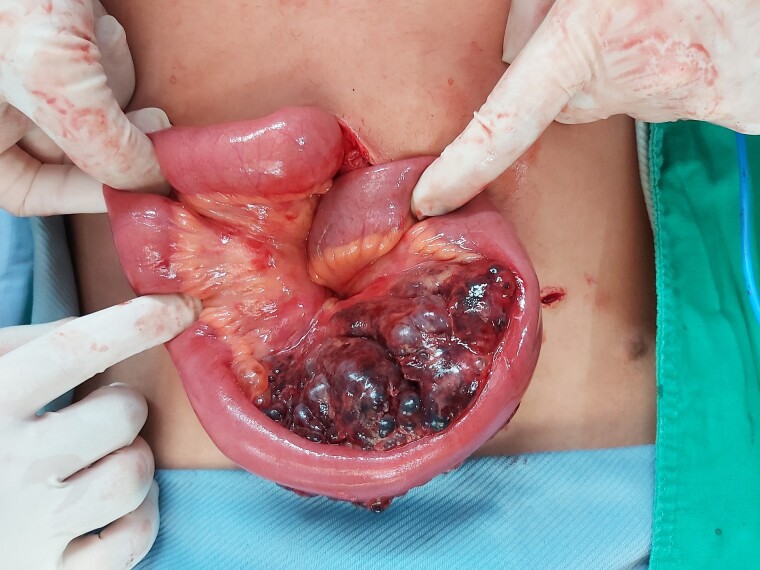# Mesenteric ruptured lymphangioma mimicking acute appendicitis

**DOI:** 10.1093/bjs/znac416

**Published:** 2022-11-25

**Authors:** Tzu-Cheng Wen, Kuo-Hua Lin

**Affiliations:** Division of General Surgery, Department of Surgery, Changhua Christian Hospital, Changhua, Taiwan; Division of General Surgery, Department of Surgery, Changhua Christian Hospital, Changhua, Taiwan

A young patient presented with migratory abdominal pain to the right iliac fossa. They regularly participated in Judo. Acute appendicitis was suspected, but at surgery, a ruptured lobulated tumour was found. Subsequent pathological examination demonstrated mesenteric lymphangioma with massive haemorrhagic necrosis and congestion.

**Figure:**